# Supplementary material for: Vitamin Intake and Loss of Muscle Mass in Older People with Type 2 Diabetes: A Prospective Study of the KAMOGAWA-DM Cohort
Source: Nutrients. 2021 Jul 8;13(7):2335. doi: 10.3390/nu13072335 (PMC8308571; doi:10.3390/nu13072335)
Supplement: Supplementary file 1 [file nutrients-13-02335-s001.zip › nutrients-1241045-supplementary.pdf]

Supplementary material

Table S1. Odds ratio of micronutrients intake on the incident muscle mass loss according to habit of exercise, sex, energy intake, protein intake, usage of supplements, and usage of biguanide.

	**Non-exerciser** ***n*** **= 85**		**Exerciser** ***n*** **= 112**	
	**OR (95% CI)**	***p***	**OR (95% CI)**	***p***
Vitamin A (μg RAE/day)	1.00 (1.00–1.00)	0.222	1.00 (1.00–1.00)	0.503
Vitamin E (mg/day)	1.03 (0.81–1.29)	0.825	0.90 (0.70–1.16)	0.426
Vitamin B1 (mg/day)	2.90 (0.14-61.80)	0.496	0.01 (0.005-0.341)	0.009
Vitamin B2 (mg/day)	1.10 (0.17-6.89)	0.922	0.83 (0.19-3.70)	0.807
Vitamin B6 (mg/day)	1.63 (0.24–11.30)	0.619	0.21 (0.03–1.24)	0.085
Vitamin B12 (μg/day)	0.94 (0.83–1.06)	0.330	0.91 (0.81–1.01)	0.087
Vitamin C (mg/day)	1.00 (1.00–1.01)	0.294	1.00 (0.99–1.01)	0.461
Vitamin D (μg/day)	0.92 (0.85–0.99)	0.029	0.92 (0.86–0.98)	0.014
	**Men** ***n*** **= 112**		**Women** ***n*** **= 85**	
	**OR (95% CI)**	***p***	**OR (95% CI)**	***p***
Vitamin A (μg RAE/day)	1.00 (1.00–1.00)	0.159	1.00 (1.00–1.00)	0.513
Vitamin E (mg/day)	1.05 (0.85–1.31)	0.659	1.00 (0.74–1.35)	0.991
Vitamin B1 (mg/day)	1.59 (0.10-24.90)	0.743	0.04 (0.001-1.35)	0.073
Vitamin B2 (mg/day)	1.93 (0.47-8.01)	0.363	0.34 (0.04-2.90)	0.322
Vitamin B6 (mg/day)	1.00 (0.20–5.03)	0.999	0.35 (0.04–3.27)	0.355
Vitamin B12 (μg/day)	0.93 (0.61–46.20)	0.132	0.94 (0.82–1.06)	0.305
Vitamin C (mg/day)	1.01 (1.00–1.01)	0.204	1.00 (0.99–1.01)	0.758
Vitamin D (μg/day)	0.95 (0.89–1.01)	0.099	0.87 (0.79–0.95)	0.003
	**Non-smoker** ***n*** **= 169**		**Smoker** ***n*** **= 28**	
	**OR (95% CI)**	***p***	**OR (95% CI)**	***p***
Vitamin A (μg RAE/day)	1.00 (1.00–1.00)	0.771	1.00 (1.00–1.00)	0.324
Vitamin E (mg/day)	0.97 (0.80–1.18)	0.763	0.89 (0.58–1.37)	0.603
Vitamin B1 (mg/day)	0.17 (0.02-1.55)	0.115	3.52 (0.01-1790.00)	0.692
Vitamin B2 (mg/day)	1.02 (0.29-3.52)	0.978	0.64 (0.03-10.18)	0.765
Vitamin B6 (mg/day)	0.46 (0.11–1.96)	0.295	0.76 (0.02–28.10)	0.880
Vitamin B12 (μg/day)	0.93 (0.84–1.02)	0.106	0.84 (0.67–1.06)	0.142
Vitamin C (mg/day)	1.00 (0.99–1.01)	0.786	1.01 (0.99–1.03)	0.629
Vitamin D (μg/day)	0.41 (0.20–0.84)	0.014	1.28 (0.03–50.23)	0.897
	**Inadequate energy intake** ***n*** **= 103**		**Adequate energy intake** ***n*** **= 94**	
	**OR (95% CI)**	***p***	**OR (95% CI)**	***p***
Vitamin A (μg RAE/day)	1.00 (1.00–1.00)	0.118	1.00 (1.00–1.00)	0.679
Vitamin E (mg/day)	0.95 (0.74–1.22)	0.710	1.02 (0.80–1.30)	0.873
Vitamin B1 (mg/day)	0.13 (0.001-10.60)	0.359	0.52 (0.05-5.88)	0.596
Vitamin B2 (mg/day)	1.02 (0.12-8.47)	0.986	1.07 (0.27-4.26)	0.920
Vitamin B6 (mg/day)	0.23 (0.02–2.64)	0.240	0.87 (0.18–4.15)	0.864
Vitamin B12 (μg/day)	0.82 (0.70–0.95)	0.011	0.99 (0.91–1.09)	0.054
Vitamin C (mg/day)	1.01 (0.99–1.02)	0.365	1.00 (0.99-.101)	0.873
Vitamin D (μg/day)	0.89 (0.83–0.97)	0.005	0.95 (0.90–1.02)	0.137
	**Inadequate protein intake** ***n*** **= 98**		**Adequate protein intake** ***n*** **= 99**	
	**OR (95% CI)**	***p***	**OR (95% CI)**	***p***
Vitamin A (μg RAE/day)	1.00 (1.00–1.00)	0.085	1.00 (1.00–1.00)	0.650
Vitamin E (mg/day)	1.07 (0.81–1.41)	0.649	0.99 (0.79–1.25)	0.949
Vitamin B1 (mg/day)	0.73 (0.01-60.90)	0.887	0.53 (0.05-5.72)	0.603
Vitamin B2 (mg/day)	4.58 (0.44-48.10)	0.204	0.97 (0.25-3.79)	0.965
Vitamin B6 (mg/day)	0.55 (0.05–6.04)	0.621	0.99 (0.21–4.66)	0.988
Vitamin B12 (μg/day)	0.92 (0.79–1.07)	0.277	0.94 (0.85–1.03)	0.199
Vitamin C (mg/day)	1.01 (1.00–102)	0.276	1.00 (0.99–1.01)	0.947

Vitamin D (µg/day)	0.88 (0.79–0.98)	0.020	0.93 (0.88–0.99)	0.017
	**Non-supplement intake** *n* = 147		**Supplement intake** *n* = 50	
	**OR (95% CI)**	***p***	**OR (95% CI)**	***p***
Vitamin A (µg RAE/day)	1.00 (1.00–1.00)	0.574	1.00 (1.00–1.00)	0.915
Vitamin E (mg/day)	1.02 (0.85–1.23)	0.830	0.77 (0.52–1.14)	0.188
Vitamin B1 (mg/day)	0.24 (0.02-2.71)	0.249	0.23 (0.003-19.60)	0.519
Vitamin B2 (mg/day)	1.10 (0.32-3.81)	0.875	0.69 (0.05-9.28)	0.779
Vitamin B6 (mg/day)	0.93 (0.21–4.19)	0.922	0.14 (0.01–2.48)	0.180
Vitamin B12 (µg/day)	0.96 (0.88–1.05)	0.368	0.71 (0.53–0.94)	0.016
Vitamin C (mg/day)	1.00 (0.99–1.01)	0.978	1.00 (0.99–1.02)	0.712
Vitamin D (µg/day)	0.93 (0.87–0.98)	0.011	0.88 (0.78–0.99)	0.032
	**Non-usage of biguanide** *n* = 132		**Usage of biguanide** *n* = 65	
	**OR (95% CI)**	***p***	**OR (95% CI)**	***p***
Vitamin A (µg RAE/day)	1.00 (1.00–1.00)	0.593	1.00 (1.00–1.00)	0.361
Vitamin E (mg/day)	0.98 (0.80–1.20)	0.853	0.95 (0.69–1.31)	0.741
Vitamin B1 (mg/day)	0.49 (0.04-5.34)	0.554	0.06 (0.001-3.77)	0.186
Vitamin B2 (mg/day)	0.75 (0.19-3.00)	0.686	0.91 (0.14-6.07)	0.919
Vitamin B6 (mg/day)	0.38 (0.09–1.59)	0.184	2.90 (0.16–52.10)	0.470
Vitamin B12 (µg/day)	0.88 (0.79–0.97)	0.013	1.04 (0.90–1.21)	0.569
Vitamin C (mg/day)	1.00 (0.995–1.01)	0.625	0.997 (0.985–1.01)	0.652
Vitamin D (µg/day)	0.91 (0.86–0.97)	0.002	0.95 (0.87–1.04)	0.232

Adjusted for age, sex, body mass index, energy intake, and protein intake. Adequate energy intake was defined energy intake ≥ 30 kcal/IBW/day, and inadequate energy intake was < 30 kcal/IBW/day. Adequate protein intake was defined as protein intake was ≥ 1.2 g/BW/day, and inadequate protein intake was defined as protein intake was < 1.2 g/BW/day.
